# Neurology

**Published:** 1897-03

**Authors:** William C. Krauss

**Affiliations:** Professor of nervous diseases, Niagara University


### NEUROLOGY.

CONDUCTED BY WILLIAM C. KRAUSS, M. D.,
Professor of nervous diseases, Niagara University.

### THE ALTERATIONS OF THE NERVE ELEMENTS IN EXPERIMENTAL UREMIC DYSCRASIA.

SACERDOTTI AND D. OTTOLENGHI, of Torino, have made very interesting experiments on the effects of uremic poisoning upon the various nerve tissues, and have published their results in the January, 1897, number of the *Rivista di Patologia Nervosa e Mentale.*

Their experiments were made on four dogs and three rabbits. In three dogs the kidneys were extirpated at one time ; in the fourth, seventy-five days intervened before extirpation. In one rabbit the kidneys were removed and in the other two the ureters were ligated near their entrance into the bladder. Of the dogs, one lived four days, two hours ; the second, five days, nine hours ; the third, seven days, ten hours ; the fourth, four days, ten hours. The rabbit whose kidneys were removed lived fifty hours, the others forty-eight and fifty-six hours respectively.

The microscopic examination of the brains of these animals offered nothing of importance. A certain amount of hyperemia was present in two of the dogs' brains, while in the other two the brains were anemic. The brain of the dog that lived seven days showed a slight diffuse opacity of the pia and a slight amount of softening of the superficial strata of the cortex. The rabbits' brains showed nothing appreciable. The methods employed were those of Golgi and Nissl.

The ganglion cells of the brain cortex of the hippocampus, cerebellum and the neuroglia cells showed a degeneration, or rather a varicose atrophy of the protoplasmic prolongations in various degrees. The authors draw the following conclusions from their experiments :

1. That ligation of the ureters or bilateral nephrectomy provokes in the nerve centers lesions easily demonstrated by Golgi's method : lesions characterised by a varicose atrophy of the dendrites of the ganglion cells, while the axis cylinder prolongation remains unaltered ; also, that, contrary to the teachings of Acquisto and Pusateri, the neuroglia cells do participate, showing varicose degeneration of the prolongations of these cells.

2. That, regarding the distribution of these lesions—(*a*) The elements, having undergone degeneration, are most diffuse in the whole cerebral cortex and appertain to the different layers of cells; (*b*) less numerous than in the cerebral cortex, but yet abundant in the altered cells in the foot of the hippocampus ; (*c*) in the cerebellum the degenerated cells are found in the molecular stratum ; (*d*) the neuroglia in the whole region studied is more or less altered.

---

### THREE CASES OF BASEDOW'S DISEASE SURGICALLY TREATED.

TRICOMI (in *Policlinico*, Fas. 8, 1896,) reports three cases of Basedow's disease, in which he made a partial resection of both lobes of the thyroid gland. As a result, the exophthalmus disappeared, cessation of the tachycardia, of the diarrhea, of the profuse perspiration, pruritus and other symptoms, increase of strength and weight of patient, and the like. The author believes that the results obtained warrant surgical procedure when the ordinary medical treatment fails to bring relief.

---

### SECONDARY DEGENERATIONS FOLLOWING FOCAL HEMORRHAGES.

G. DOTTO AND E. PUSATERI, of Palermo, have undertaken to study the alterations in the nerve cells of the cerebral cortex secondary to intracerebral focal hemorrhages, and upon the connection between the cortex of the Isle of Reil with the capsula externa in man (*Rivista di Patologia Nervosa e Mentale*, January, 1897).

As a result of their studies they feel warranted in drawing the following conclusions:

1. Following intracerebral focal hemorrhages there follows a secondary atrophic process in the cerebral cortex of the hemisphere of the same side.

2. That these alterations are not uniformly diffuse, and interest in different degrees the nerve elements.

3. That the cortex of the Isle of Reil in man *is* in connection with the external capsule.

---

### ABOLITION OF THE REFLEX OF THE TENDON OF ACHILLES IN SCIATICA.

J. Babinsky (*Gazette des Hôpitaux*, N. 150, 1896,) shows that in healthy individuals the tendon reflex of the Achilles tendon is normal, while in disturbances of the sciatic nerve the reflex is either abolished or greatly diminished. He found this phenomenon not only in cases of intense sciatica with marked muscular atrophy (sciatic neuritis), but also in the lighter forms of ischialgia, designated sciatic neuralgia.

The author refers to two cases where the difference between the behavior of the Achilles tendon reflex on the well side and the affected side was very conspicuous, being totally abolished on the affected side, while on the unaffected side it was normal. This sign, the author concludes, is a valuable diagnostic aid ; it indicates the existence of some organic change in the nerve and excludes the hypothesis of simulation, also aids in differentiating between a true and an hysterical sciatica. In the latter, according to Babinsky, the symptom would be wanting.

---

### CONTRIBUTIONS TO THE CLINICAL AND PATHOLOGICAL STUDY OF ACROMEGALY.

Dr. E. Comini (in the *Archivio per le Scienze Mediche*, Vol. XX., Fas. 4, 1896,) reports three cases of acromegaly. In the first case the objective symptoms of acromegaly are pronounced, the only subjective symptom being a slight morning headache. In the second case there is disturbance of vision, insomnia, symptoms developed rapidly—disturbance of sensation about the arms ; marked improvement followed the use of thyroidine. In the third case muscular atrophy was present along with a peripheral neuritis. The autopsy on this case showed congestion of the pia ; cerebral edema ; the pituitary gland enlarged so as to resemble a pigeon's egg, and softened, the posterior part of the sella turcica narrowed and deformed ; normal size of the thyroid gland, weight gr. 100 ; no trace of persistence of the thymus gland ; atrophy of the muscles of the forearms and hands, which stood in contrast to the hypertrophy of the corresponding bones. Microscopical examination showed histological alterations in the hypophysis, in the thyroid and a neuritis of the radial nerves.

---

THYROIDECTOMY FOR RELIEF OF EXOPHTHALMIC GOITRE.

Dr. Antonio Gandalfo (*Anales del Circulo Med. Argentino*, October 15, 1896,) reports a case of Basedow's disease in a patient thirty-three years old, who had noticed a swelling of the neck thyroid for the past eight years.

On entrance to hospital at Buenos Ayres a large bilateral tumor of the thyroid was found, more voluminous on the right side, with exophthalmus, Graefe's symptom, enlargement of the heart with mitral direct murmur, tachycardia (110-130), trill in the carotids, memory is poor, is extremely excitable, has flushings—but no chills, skin is pale, anemic ; the whole body trembles ; operated on August 2, 1895. Ten months after the operation his condition was found to be as follows : the thyroid enlargement has, of course, disappeared, the exophthalmus has diminished, especially on the right side ; Graefe's sign no longer exists, while vision is better and clearer ; the heart is still increased in volume, its tones are clear and regular during the day, but at night become irregular, sometimes intermittent ; no murmurs ; pulse is regular, 90-100 pulsations per minute. The sensations of heat, perspiration, the tremor and irritability have disappeared and the patient is regarded as greatly improved.

---

THE FORMS OF DIABETES.

Dr. George Harley (*Indiana Lancet—Lancet-Clinic*) gives the following classification of diabetes :

1. Hepatic diabetes—including the gouty variety.

2. Cerebral diabetes—including all cases of saccharine urine arising from nerve derangements.

3. Pancreatic diabetes —the most deadly form of the disease.

4. Hereditary diabetes—a form by no means uncommon, and one, too, where both brothers and sisters may labor under the disease without either their maternal or paternal having been affected by diabetes, though more distant members of the family may have suffered from it.

5. Food diabetes—including all forms of saccharine urine arising from the ingestion of unwholesome substances.

In the matter of treatment, besides diet and opium or codeine, Dr. Harley recommends croton chloral, strychnine, phosporic acid for thirst, and an absolute prohibition of alcohol.

